# Quantitative anatomy of the primary ossification center of the femoral shaft in human fetuses

**DOI:** 10.1007/s00276-017-1861-8

**Published:** 2017-04-25

**Authors:** Mariusz Baumgart, Marcin Wiśniewski, Magdalena Grzonkowska, Mateusz Badura, Bogdan Małkowski, Michał Szpinda

**Affiliations:** 1 Department of Normal Anatomy, The Ludwik Rydygier Collegium Medicum in Bydgoszcz, The Nicolaus Copernicus University in Toruń, Łukasiewicza 1 Street, 85-821 Bydgoszcz, Poland; 2 Department of Positron Emission Tomography and Molecular Imaging, The Ludwik Rydygier Collegium Medicum in Bydgoszcz, The Nicolaus Copernicus University in Toruń, Łukasiewicza 1 Street, 85-821 Bydgoszcz, Poland

**Keywords:** Femur, Primary ossification center, Size, Growth dynamics, Human fetus

## Abstract

**Purpose:**

Early clinical distinction of congenital defects in the femur is extremely important, as it determines the prognosis of the development of the lower limb. This study was performed to quantitatively examine the primary center of ossification in the femoral shaft with respect to its linear, planar, and volumetric parameters.

**Materials and methods:**

Using methods of CT, digital-image analysis, and statistics, the size of the primary ossification center of the femoral shaft in 47 spontaneously aborted human fetuses aged 17–30 weeks was studied.

**Results:**

With no sex and laterality differences, the best fit growth dynamics for femoral shaft ossification center was modelled by the following functions: *y* = 5.717 + 0.040 × (age)^2^ ± 2.905 (*R* ^2^ = 0.86) for its length, *y* = −3.579 + 0.368 × age ± 0.529 (*R* ^2^ = 0.88) for its proximal transverse diameter, *y* = −1.105 + 0.187 × age ± 0.309 (*R* ^2^ = 0.84) for its middle transverse diameter, *y* = −2.321 + 0.323 × age ± 0.558 (*R* ^2^ = 0.83) for its distal transverse diameter, *y* = −50.306 + 0.308 × (age)^2^ ± 18.289 (*R* ^2^ = 0.90) for its projection surface area, and *y* = −91.458 + 0.390 × (age)^3^ ± 92.146 (*R* ^2^ = 0.88) for its volume.

**Conclusions:**

The size of the femoral shaft ossification center displays neither sex nor laterality differences. The ossification center in the femoral shaft follows quadratic functions with respect to its length and projection surface area, linear functions with respect to its proximal, middle, and distal transverse diameters, and a cubic function with respect to its volume. The obtained morphometric data of the femoral shaft ossification center are considered normative for respective prenatal weeks and may be of relevance in both the estimation of fetal ages and the ultrasound diagnostics of congenital defects.

## Introduction

In the first trimester of pregnancy, the most precise method in determining fetal ages is the crown-rump length [2]. However, in the second and third trimesters, femur length measurement is one of the standards in the morphometric assessment of human fetuses in terms of their growth and maturity, as well as detection of their congenital defects [21].

Non-invasive ultrasound examinations usually include length measurements of the primary ossification center in the femoral shaft, as the two epiphyseal cartilaginous parts are not well visualized [14]. Of note, using ultrasound examination, ossification centers can be discernible if their dimensions exceed 1 mm [12]. Harcke et al. [11] performed ultrasound and radiographic examinations of the femoral heads to compare the accuracy of both modalities, and demonstrated a much better precision of ultrasound examinations, with ossification centers noticed in 343 cases when compared to 292 cases for radiographic imaging.

Early clinical distinction of congenital defects of the femur is extremely important, as it determines the prognosis of the development of the lower limb. Femoral defects often coincide with skeletal defects and include hypoplasia or complete absence (agenesis) of the femur, while in rare cases, they may accompany the defects of the central nervous system, thorax, and abdomen [18].

In the present study, we aimed:

- to perform morphometric analysis of linear, planar, and spatial parameters of the femoral shaft ossification center in human fetuses to determine their normative values;
- to establish possible differences between sexes for all analyzed parameters;
- to compute growth dynamics for all the analyzed parameters, expressed by best-matched mathematical models.

## Materials and methods

The study material comprised 47 human fetuses of both sexes (25 males and 22 females) aged 17–30 weeks, originating from spontaneous abortions or preterm deliveries. The material was acquired before the year 2000 and remains part of the specimen collection of our Department of Normal Anatomy. The experiment was approved by the Bioethics Committee of our University (KB 275/2011). The fetal age was determined based on the crown-rump length. Table 1 lists the characteristics of the study group, including age, number, and sex of the fetuses studied.

**Table 1:** Age, number, and sex of the fetuses studied

Gestational age (weeks)	Crown-rump length (mm)				Number of fetuses	Sex	
	Mean	SD	Min.	Max.		♂	♀
17	116.00	1.41	115.00	117.00	2	1	1
18	130.00	0.00	130.00	130.00	2	1	1
19	150.00	3.03	146.00	154.00	6	3	3
20	159.50	0.71	159.00	160.00	2	1	1
21	174.75	2.87	171.00	178.00	4	3	1
22	184.67	1.53	183.00	186.00	3	1	2
23	197.75	2.99	195.00	202.00	4	3	1
24	208.57	3.74	204.00	213.00	7	4	3
25	214.50	0.71	214.00	215.00	2	1	1
26	226.00	1.41	225.00	227.00	2	1	1
27	237.75	2.75	235.00	241.00	4	3	1
28	246.67	4.93	241.00	250.00	3	1	2
29	254.00	1.41	253.00	255.00	2	1	1
30	263.25	1.26	262.00	265.00	4	1	3
Total					47	25	22

**Fig. 1:**
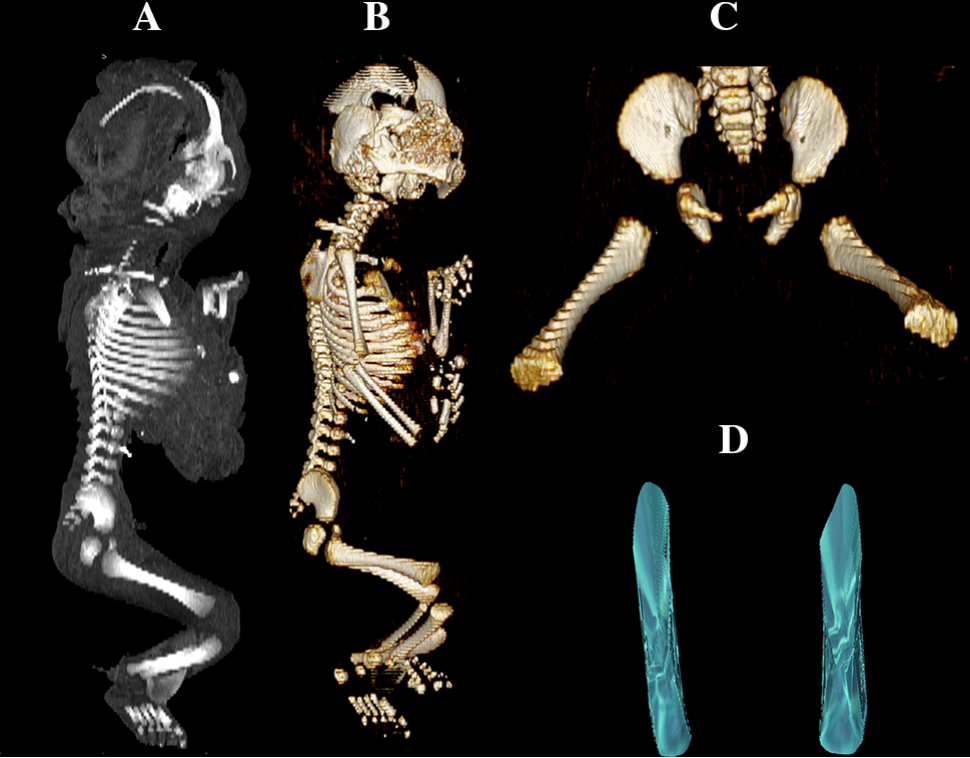
CT of a female fetus aged 27 weeks (in the sagittal projection) recorded in DICOM formats (**a**), with the sagittal reconstruction of its bones (**b**), with the frontal projection of its pelvic girdle and right and left femurs (**c**), and with 3D reconstructions of its right and left femoral shaft ossification centers (**d**) assessed by Osirix 3.9

**Fig. 2:**
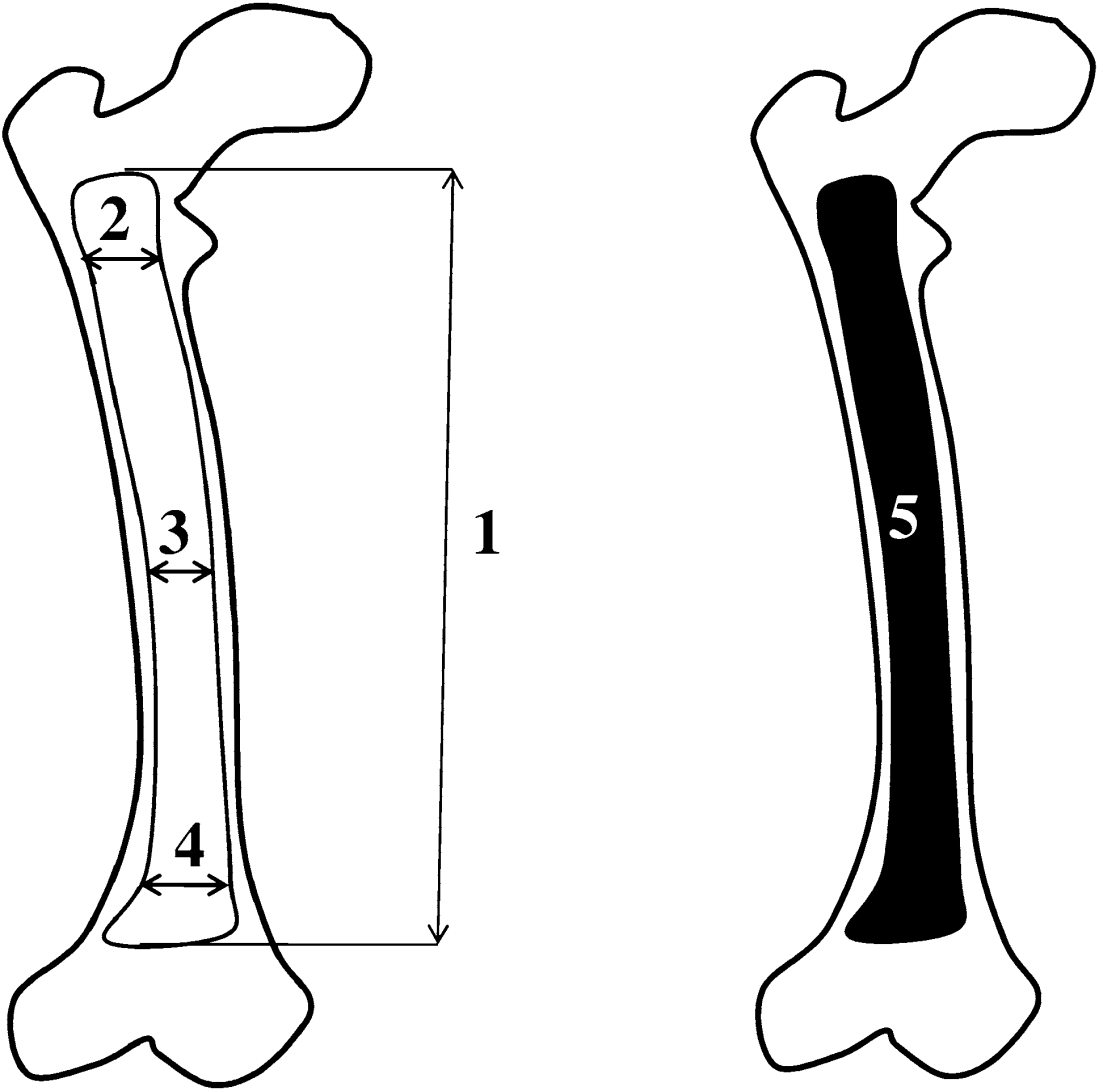
Diagram showing measurements of the femoral shaft ossification center in the horizontal projection: *1* length, *2* proximal transverse diameter, *3* middle transverse diameter, *4* distal transverse diameter, and *5* projection surface area

Using the Siemens-Biograph 128 mCT scanner, scans of fetuses in DICOM formats were acquired at 0.4 mm intervals (Fig. 1). Measurements for each femur were conducted in a specific sequence, as specified in Fig. 2. In each fetus, the assessment of linear diameters, projection surface area, and volume of the femoral shaft ossification center was carried out. Despite a cartilaginous stage of the proximal and distal ends of the femur, a morphometric analysis regarding their transverse and sagittal dimensions and volume was feasible, as the contours of the proximal and distal ends were already clearly visible [4, 6].

On the right and left sides, measurements of the femoral shaft ossification center included the following six parameters:

1. length, based on the determined distance between the proximal and distal borderlines of the ossification center in the frontal plane (Fig. 2);
2. proximal transverse diameter, based on the determined distance between the medial and lateral borderlines of the proximal region of the ossification center in the frontal plane (Fig. 2);
3. middle transverse diameter, based on the determined distance between the medial and lateral borderlines of the central region of the ossification center in the frontal plane (Fig. 2);
4. distal transverse diameter, based on the determined distance between the medial and lateral borderlines of the distal region of the ossification center in the frontal plane (Fig. 2);
5. projection surface area, based on the determined contour of the femoral shaft ossification center in the frontal plane (Fig. 2);
6. volume, calculated using advanced diagnostic imaging tools for 3D reconstruction, taking into account position and the absorption of radiation by bone tissue (Fig. 1d).

All measurements were performed by one researcher (M.B). Each measurement was performed three times under the same conditions but at different times, and averaged. The differences between the repeated measurements as the intra-observer variation were assessed by the one-way ANOVA test for paired data and post hoc RIR Tukey test. The numerical data were statistically analyzed. Distribution of variables was checked using the Shapiro–Wilk (W) test, while the homogeneity of variance was checked using Fisher’s test. The results were expressed as arithmetic means with standard deviations (SD). To compare the means, Student’s *t* test for independent variables and one-way ANOVA were used. Tukey’s test was used for post hoc analysis. If no similarity of variance occurred, the non-parametric Kruskal–Wallis test was used. The characterization of developmental dynamics of the analyzed parameters was based on linear and curvilinear regression analysis. The match between the estimated curves and measurement results was evaluated based on the coefficient of determination (*R* ^2^).

## Results

No statistically significant difference was found in evaluating intra-observer reproducibility of the measurements of the femoral shaft ossification center. Mean values and standard deviations of the analyzed parameters of the left and right femoral shaft ossification centers in human fetuses at varying gestational ages are presented in Tables 2 and 3 for length and proximal, middle, and distal transverse diameters and in Table 4 for projection surface area and volume.

**Table 2:** Length and transverse diameters for: proximal end, middle part, and distal end of the right femoral shaft ossification center in human fetuses

Gestational age (weeks)	Number of fetuses	Ossification center of the right femur							
		Length (mm)		Transverse diameter (mm)					
				Proximal end		Middle part		Distal end	
		Mean	SD	Mean	SD	Mean	SD	Mean	SD
17	2	18.29	0.71	2.92	0.08	2.02	0.07	3.11	0.21
18	2	19.63	2.23	2.92	0.35	2.18	0.13	3.41	0.06
19	6	19.66	6.03	3.25	0.45	2.44	0.27	3.61	0.26
20	2	22.83	4.45	3.90	0.09	2.52	0.49	4.34	0.33
21	4	23.65	2.63	4.17	0.53	2.77	0.39	4.56	0.79
22	3	25.17	2.15	4.28	0.60	2.81	0.42	4.61	0.47
23	4	27.30	3.45	4.31	0.40	2.96	0.24	4.81	0.47
24	7	29.08	4.44	5.46	0.43	3.56	0.26	5.43	0.51
25	2	31.04	1.41	6.09	0.08	3.78	0.08	5.64	0.08
26	2	31.74	1.41	6.10	0.54	3.94	0.35	6.33	0.12
27	4	35.42	2.23	6.40	0.40	3.99	0.08	6.67	0.80
28	3	36.18	1.41	6.67	0.43	4.05	0.31	6.77	0.73
29	2	37.23	3.37	6.74	0.63	4.55	0.30	6.91	0.05
30	4	41.35	2.65	7.66	0.25	4.85	0.15	7.15	0.57

**Table 3:** Length and transverse diameters for: proximal end, middle part, and distal end of the left femoral shaft ossification center in human fetuses

Gestational age (weeks)	Number of fetuses	Ossification center of the left femur							
		Length (mm)		Transverse diameter (mm)					
				Proximal end		Middle part		Distal end	
		Mean	SD	Mean	SD	Mean	SD	Mean	SD
17	2	15.96	0.00	2.69	0.49	2.16	0.14	3.04	0.14
18	2	16.01	0.88	2.94	0.40	2.38	0.10	3.26	0.13
19	6	19.15	2.09	3.25	0.41	2.45	0.15	3.70	0.38
20	2	21.69	2.75	3.93	0.17	2.51	0.17	4.25	0.13
21	4	22.03	2.29	3.98	0.67	2.83	0.28	4.37	0.54
22	3	25.44	1.95	4.09	0.36	2.96	0.29	4.70	0.67
23	4	27.54	3.92	4.28	0.35	3.01	0.10	4.95	0.78
24	7	29.90	3.90	5.49	0.65	3.31	0.27	5.37	0.16
25	2	31.43	1.41	6.09	0.27	3.64	0.10	5.53	0.59
26	2	31.61	1.98	6.36	0.44	3.81	0.06	6.43	0.13
27	4	36.40	2.81	6.43	0.43	3.90	0.36	6.43	0.91
28	3	36.68	4.55	6.63	0.47	4.19	0.24	6.44	0.62
29	2	38.14	1.41	7.00	0.22	4.27	0.43	6.67	0.13
30	4	39.72	3.61	7.59	0.32	4.33	0.26	7.19	0.56

**Table 4:** Projection surface area and volume of the femur`s shaft ossification center

Gestational age	Number of fetuses	Ossification center of femur							
		Projection surface area (mm^2^)				Volume (mm^3^)			
		Right femur		Left femur		Right femur		Left femur	
		Mean	SD	Mean	SD	Mean	SD	Mean	SD
17	2	42.90	1.41	43.10	0.71	133.33	1.41	129.97	28.64
18	2	44.80	10.18	47.00	8.20	153.50	13.28	151.25	10.69
19	6	57.45	13.26	57.08	14.26	158.80	30.56	155.80	2.83
20	2	71.65	15.63	80.45	14.64	233.55	91.29	256.95	10.68
21	4	81.13	24.24	83.55	26.18	249.17	99.15	278.90	40.69
22	3	94.20	14.23	89.13	13.81	283.53	47.12	286.90	91.27
23	4	100.28	20.83	107.43	11.98	359.43	34.18	362.88	21.87
24	7	121.86	25.65	121.57	24.66	460.30	15.11	451.20	3.54
25	2	128.41	1.56	136.81	2.12	482.23	7.07	484.59	14.58
26	2	164.30	4.95	166.40	1.41	500.05	2.90	486.80	14.14
27	4	179.45	18.09	178.64	28.81	652.03	57.65	696.05	14.08
28	3	183.10	10.61	179.68	10.65	734.70	4.24	731.00	7.78
29	2	193.63	28.73	205.63	2.83	800.67	12.39	800.80	17.43
30	4	220.80	22.81	214.18	15.72	932.20	12.51	939.65	12.28

The statistical analysis revealed neither significant sex nor bilateral differences. This allowed us to compute one growth curve for each analyzed parameter.

**Fig. 3:**
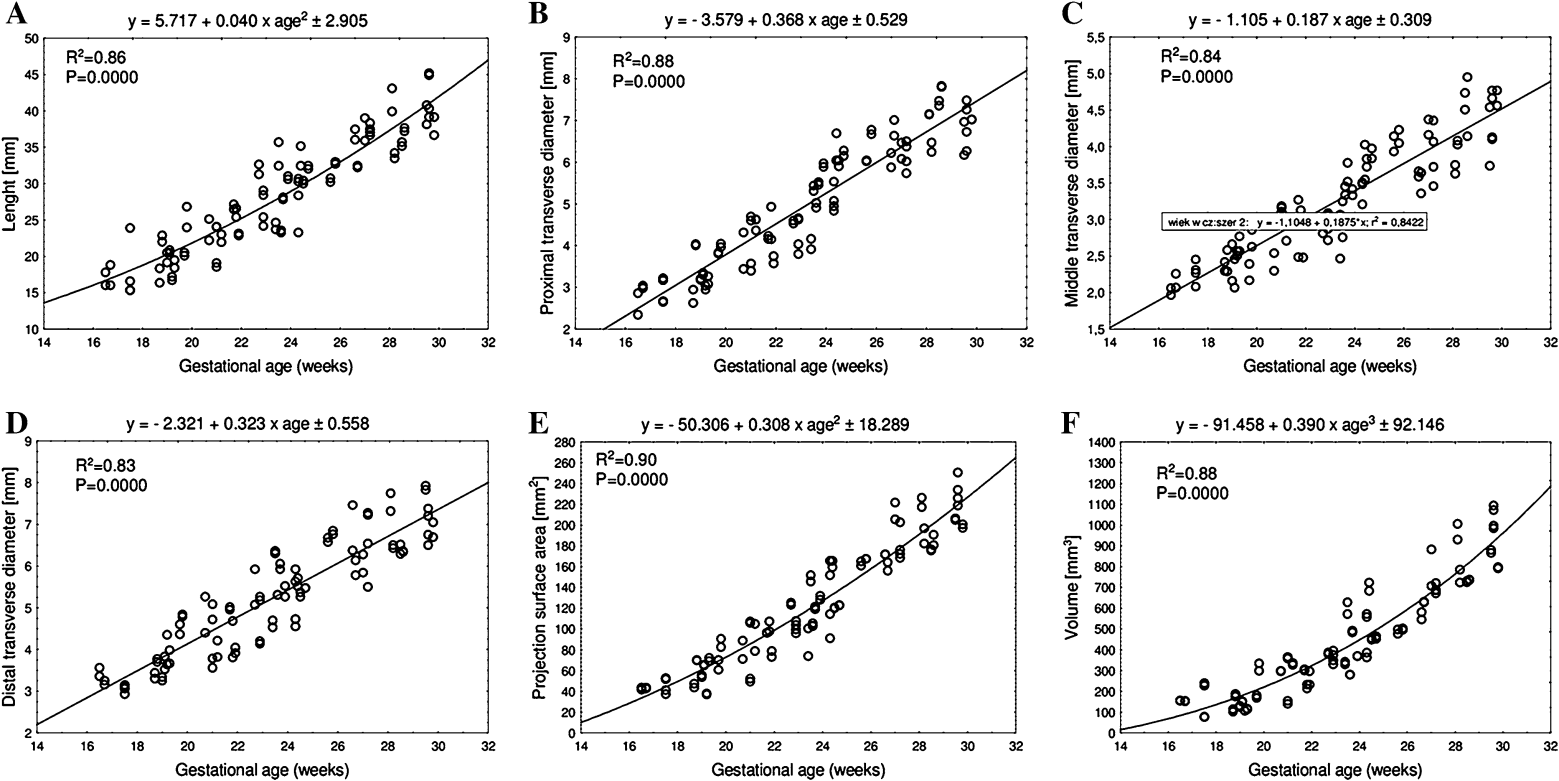
Regression lines for length (**a**), proximal (**b**), middle (**c**) and distal (**d**) transverse diameters, projection surface area (**e**), and volume (**f**) of the femoral shaft ossification center

The mean length of the femoral shaft ossification center at fetal ages of 17–30 weeks grew from 18.29 ± 0.71 to 41.35 ± 2.65 mm on the right and from 15.96 ± 0.00 to 39.72 ± 3.61 mm on the left, following the quadratic function *y* = 5.717 + 0.040 × (age)^2^ ± 2.905 (*R* ^2^ = 0.86)—(Fig. 3a).

The mean proximal transverse diameter of the femoral shaft ossification center at fetal ages of 17–30 weeks grew from 2.92 ± 0.08 to 7.66 ± 0.25 mm on the right and from 2.69 ± 0.49 to 7.59 ± 0.32 mm on the left, following the linear function *y* = −3.579 + 0.368 × age ± 0.529 (*R* ^2^ = 0.88)—(Fig. 3b). The mean middle transverse diameter of the femoral shaft ossification center at fetal ages of 17–30 weeks grew from 2.02 ± 0.07 to 4.85 ± 0.15 mm on the right and from 2.16 ± 0.14 to 4.33 ± 0.26 mm on the left, in accordance with the linear function *y* = −1.105 + 0.187 × age ± 0.309 (*R* ^2^ = 0.84)—(Fig. 3c). In fetuses aged 17–30 weeks, the mean distal transverse diameter of the femoral shaft ossification center ranged from 3.11 ± 0.21 to 7.15 ± 0.57 mm on the right, and from 3.04 ± 0.14 to 7.19 ± 0.56 mm on the left, following the linear function: *y* = −2.321 + 0.323 × age ± 0.558 (*R* ^2^ = 0.83)—(Fig. 3d).

The mean projection surface area of the femoral shaft ossification center ranged from 42.90 ± 1.41 mm^2^ at 17 weeks to 220.80 ± 22.81 mm^2^ at 30 weeks on the right and from 43.10 ± 0.71 to 214.18 ± 15.72 mm^2^, respectively, on the left, following the quadratic function *y* = −50.306 + 0.308 × (age)^2^ ± 18.289 (*R* ^2^ = 0.90)—(Fig. 3e).

The mean volume of the right and left femoral shaft ossification centers in the fetal age range of 17–30 weeks increased from 133.33 ± 1.41 to 932.20 ± 12.51 mm^3^ on the right and from 129.97 ± 28.64 to 939.65 ± 12.28 mm^3^ on the left, following the cubic function: *y* = −91.458 + 0.390 × (age)^3^ ± 92.146 (*R* ^2^ = 0.88)—(Fig. 3f).

## Discussion

Of all long bones, the femur is the second one—just after the clavicle—in which the process of ossification starts [1]. Ossification of the femur commences in the middle part of its shaft and simultaneously advances towards its both ends. Ossification of the femoral shaft and the femoral epiphyses follows in a disparate fashion. The primary ossification center appears in the middle part of the shaft at week 7 of gestation, while the secondary ossification centers located within the proximal and distal epiphyses appear at much later stages. There are three ossification centers in the proximal femoral end located in its head, greater and lesser trochanters, whereas there exists only one ossification center in the distal femoral end. The process of ossification begins in the femoral head between months 6 and 12 after birth, in the greater trochanter at year 4, and in the lesser trochanter at year 14. The complete fusion of the femoral shaft with the lesser trochanter, greater trochanter, and femoral head occurs in individuals aged 16, 17, and 18 years, respectively. The femoral neck ossifies due to the extension of the primary ossification center of the shaft [16]. Ossification of the distal femoral end begins between weeks 23 and 40 of gestation [3]. However, these findings have been queried by Panattoni et al. [15], who observed the onset of ossification in the trochanters during the intrauterine growth. Therefore, these authors hypothesized that neither extended posture nor ambulation affect, as it had previously been thought, the initiation of the development of the trochanteric ossification centers despite fetal movements can stimulate such ossification.

Having analyzed the ossification center in the distal femoral epiphysis in 140 human fetuses, Pryor [17] observed its earlier appearance in females, i.e., at the age of 25–30 weeks than in males, i.e., at the age of 30–40 weeks. The ultrasound examination by Zhianpour and Golshanara [20] revealed the ossification center in the distal femoral epiphysis in 58.8% of fetuses at 29 weeks and in all fetuses at 36 weeks. With the use of MRI, Nemec et al. [14] found the distal femoral epiphysis in single fetuses start to ossify at 25 weeks, and this process involved all fetuses aged 35 weeks. Gentili et al. [8] ultrasonically revealed the first ossification centers in the distal femoral epiphysis to be visualized as late as at week 32 and in 94.5% of fetuses at week 34. These authors also measured the diameter of the ossification center that ranged from 6 to 9 mm; however, they did not precisely report which diameter of the ossification center was taken into account. They also noted that understanding of the diameter of the ossification center in the distal femoral epiphysis allows defining fetal maturity in a much more precise way. In intrauterine growth retardation, the ossification center in the distal femoral epiphysis developed normally in only 33.3% of cases was considerably reduced in 25% of cases and did not occur at all in 41.6% of cases. The authors postulated that in fetuses lacking ossification centers in the distal femur and the proximal tibia, the fetal age was less than 32 weeks, while in those with the ossification center present in the distal femur but absent in the proximal tibia, the fetal age was between 32 and 36 weeks. Furthermore, ossification centers in both the femur and tibia were typical of fetuses older than 36 weeks. Donne et al. [5] also claimed that measurements of the ossification center in the distal femoral epiphysis enhanced precision in determining fetal ages. They demonstrated that in 96% of fetuses over the age of 32 weeks, the ossification center was observed in the distal femoral epiphysis.

There are different reports in the medical literature about the times of appearance of femoral ossification centers in different ethnic populations. Using ultrasound Birang et al. [2] measured the diameter of the distal femoral ossification center in the sagittal plane in the population of Iran. The first distal epiphyseal ossification center was already found at week 29 of gestation, in 56% of fetuses at week 33, in 96% of fetuses at week 36, and in all fetuses aged 37 weeks. In turn, according to Donne et al. [5], the first ossification center in the distal femoral epiphysis in Brazilians was visible as late as at week 30 of gestation and in all examined fetuses at week 37, incidentally as in the Iranian population. Mahony et al. [12] demonstrated in the American population that the distal epiphyseal ossification center in the femur was most frequently visible between weeks 32 and 33 of pregnancy, and the age of fetuses without this ossification center should have been estimated younger than 34 weeks. In the Chinese population, Wu et al. [19] demonstrated the distal epiphyseal ossification center in the femur starting with week 29 of gestation, with the center present in every individuals since week 34.

According to Birang et al. [2], the mean sagittal diameter of the distal epiphyseal ossification center was 0.08 ± 0.37 mm at 29 weeks, 1.26 ± 1.25 mm at 33 weeks, and 4.20 ± 1.51 mm at 37 weeks of gestation. They stated that diameters of the distal epiphyseal ossification center allowed assessing fetal ages in the third trimester of pregnancy. The sagittal diameters of 0.5 and 0.9 mm indicated the fetal ages of 30.42 ± 1.94 and 37.25 ± 0.44 weeks, respectively [2]. In turn, Goldstein et al. [10] observed that the sagittal diameter equal to or greater than 3 mm in 84% of cases indicated the fetal age over 37 weeks.

Felts [7] carried out various measurements in human fetuses, including the length of the femoral shaft ossification center, as well as the transverse and sagittal diameters of the ossified part of the shaft, both measured in their narrowest portions. An increase in length of the femoral shaft ossification center was presented as the linear function *y* = −5.57 + 0.840 × age. It was noted that the ossification center could already be observed in the femur of 6.6 mm long, and every increase in femoral length by 1 mm was accompanied by an increase in length of the ossification center by 0.84 mm. The transverse and sagittal diameters of the ossified part of the femoral shaft increased proportionately to fetal age, according to the functions: *y* = 6.8 + 0.009 × age and *y* = 7.1 + 0.014 × age, respectively. In this study, it was demonstrated that the length of the femoral shaft ossification center followed the quadratic function *y* = 5.717 + 0.040 × (age)^2^ ± 2.905. The proximal, middle, and distal transverse diameters of the femoral ossification center increased in accordance with the linear functions: *y* = −3.579 + 0.368 × age ± 0.529, *y* = −1.105 + 0.187 × age ± 0.309, and *y* = −2.321 + 0.323 × age ± 0.558, respectively.

This study has been the first to provide original data regarding the projection surface area and volume of the femoral shaft ossification center in the fetal age range of 17–30 weeks. We showed that the projection surface area and volume of the femoral shaft ossification center modelled the following functions: *y* = −50.306 + 0.308 × (age)^2^ ± 18.289 and *y* = −91.458 + 0.390 × (age)^3^ ± 92.146, respectively.

Regrettably, there have been no reports in the medical literature concerning the dimensions of the femoral shaft ossification center, which precludes a more comprehensive discussion in this subject.

The dimensions of the femoral shaft ossification center obtained in the present study may be critically useful in diagnosing skeletal dysplasias that are often characterized by a disrupted or restricted growth of fetuses. Developmental defects of the femur include proximal femoral focal deficiency, congenital short femur, and Meyer dysplasia. Proximal femoral focal deficiency is characterized by the fact that the affected femur is 35–50% of the length of a normal bone, while in congenital short femur, it is 40–60% of the length of a normal bone [9]. Meyer dysplasia is caused by a delayed and uneven development of the femoral proximal epiphyseal ossification center. This disorder is five times more frequent in boys, is usually detected at approximately 2 years of age, and usually spontaneously recedes by the age of 6 years [13].

## Conclusions

1. The size of the femoral shaft ossification center displays neither sex nor laterality differences.
2. The ossification center in the femoral shaft follows quadratic functions with respect to its length and projection surface area, linear functions with respect to its proximal, middle, and distal transverse diameters, and a cubic function with respect to its volume.
3. The obtained morphometric data of the femoral shaft ossification center are considered normative for respective prenatal weeks and may be of relevance in both the estimation of fetal ages and the ultrasound diagnostics of congenital defects.
